# Supplementary material for: Investigating the nature of open science practices across complementary, alternative, and integrative medicine journals: An audit
Source: PLoS One. 2024 May 3;19(5):e0302655. doi: 10.1371/journal.pone.0302655 (PMC11068175; doi:10.1371/journal.pone.0302655)
Supplement: Attachment — Submitted filename: Response to Reviewers_Mar1824.docx [file pone.0302655.s002.docx]

**Response to Reviewers**

Dear Editor:

Thank you for your email. I am pleased to hear that you believe that our manuscript will have the potential to be published in PLOS ONE following requested revisions.

As per your request, please find our response to the peer-reviewers’ comments with every change outlined point by point below:

=====================================================================

Journal Requirements:

When submitting your revision, we need you to address these additional requirements.

1. Please ensure that your manuscript meets PLOS ONE's style requirements, including those for file naming. The PLOS ONE style templates can be found at 
<https://journals.plos.org/plosone/s/file?id=wjVg/PLOSOne_formatting_sample_main_body.pdf> and 
<https://journals.plos.org/plosone/s/file?id=ba62/PLOSOne_formatting_sample_title_authors_affiliations.pdf>

2. Please upload a copy of Figure 1, to which you refer in your text on page 8. If the figure is no longer to be included as part of the submission please remove all reference to it within the text.

Additional Editor Comments:

I agree with both reviewers about the need for revisions of the manuscript.

- We kindly thank the editor for providing their feedback on our manuscript.

[Note: HTML markup is below. Please do not edit.]

Reviewers' comments:

Reviewer's Responses to Questions

**Comments to the Author**

1. Is the manuscript technically sound, and do the data support the conclusions?

The manuscript must describe a technically sound piece of scientific research with data that supports the conclusions. Experiments must have been conducted rigorously, with appropriate controls, replication, and sample sizes. The conclusions must be drawn appropriately based on the data presented.

Reviewer #1: Partly

Reviewer #2: Yes

2. Has the statistical analysis been performed appropriately and rigorously?

Reviewer #1: N/A

Reviewer #2: Yes

3. Have the authors made all data underlying the findings in their manuscript fully available?

The [PLOS Data policy](http://www.plosone.org/static/policies.action#sharing) requires authors to make all data underlying the findings described in their manuscript fully available without restriction, with rare exception (please refer to the Data Availability Statement in the manuscript PDF file). The data should be provided as part of the manuscript or its supporting information, or deposited to a public repository. For example, in addition to summary statistics, the data points behind means, medians and variance measures should be available. If there are restrictions on publicly sharing data—e.g. participant privacy or use of data from a third party—those must be specified.

Reviewer #1: Yes

Reviewer #2: Yes

4. Is the manuscript presented in an intelligible fashion and written in standard English?

PLOS ONE does not copyedit accepted manuscripts, so the language in submitted articles must be clear, correct, and unambiguous. Any typographical or grammatical errors should be corrected at revision, so please note any specific errors here.

Reviewer #1: Yes

Reviewer #2: Yes

5. Review Comments to the Author

Please use the space provided to explain your answers to the questions above. You may also include additional comments for the author, including concerns about dual publication, research ethics, or publication ethics. (Please upload your review as an attachment if it exceeds 20,000 characters)

Reviewer #1: I appreciate the author’s attention to this topic as well as the transparency in the supplemental files and also the explicit detail on the articles included. I also appreciate the specificity in the discussion that holds potential to help to advance rigor in research practices in the field. I feel it will be a valuable addition to the scholarly literature with some additional clarifications that include the following:

- We kindly thank this reviewer for providing their feedback on our manuscript.

• It would be helpful if the authors included some additional information according to PRISMA guidelines for systematic reviews such as the flow chart showing inclusions and exclusions as well as additional information on their initial screening procedures and interrater reliability: <http://www.prisma-statement.org/?AspxAutoDetectCookieSupport=1> I do note that the authors state that Figure 1 is the flow chart for inclusion/exclusion but I could not find a Figure in the PDF document for review nor the supplemental files linked. I apologize if I missed in somewhere but did try to find it and also did document searches for it.

- We would like to clarify that we conducted an audit rather than a systematic review, meaning PRISMA guidelines would not apply. However, we understand the importance of clearly describing our eligibility criteria and created that flow chart to show our process. Unfortunately, we did not include it in the initial submission, and we apologise for this confusion. We have now added the missing figure as a file to the revised submission.

• I am not an expert in contemporary and alternative medicines so I cannot verify if the eligibility criteria for particular journals would accurately capture this field of literature. Therefore, it would be helpful to readers if the authors could clarify how they came up with the criteria for inclusion/exclusion and whether: a) they consulted an expert from the field of CAIM to ensure they are accurately capturing the literature or b) if perhaps there are other systematic reviews conducted within the field of CAIM that utilized similar inclusion criteria for journals. Overall, I just would like more information verifying that the eligibility criteria accurately captured the literature of the particular field of CAIM. I put this note in because there have been other systematic reviews that have not adequately represented the field of expertise due to their inclusion/exclusion criteria; ultimately resulting in bias due to missing data. Hence, I think a few clarifying statements ensuring how this criteria adequately captures the breadth of literature of CAIM would be helpful in understanding and would ultimately mitigate these questions.

- We would like to clarify that we conducted an audit rather than a systematic review.
- We initiated the selection process using the list of CAIM journals from Ng’s bibliometric analysis since it was derived from Scopus, the largest and most relevant database for our purposes. We wanted our audit to focus only on general CAIM journals rather than journals focused on a specific CAIM therapy (ex. homeopathy), as any CAIM topic can go into a general CAIM journal but not vice versa with general CAIM research nearly never being published in those specific journals. As a result, we modified Ng’s list to only include journals with “complementary,” “alternative,” or “integrative” in their titles. We note that Dr. Holger Cramer and Dr. Jeremy Ng are experts on the subject area of CAIM, and they were involved in creating and approved of final the eligibility criteria used in this audit. We understand our reasoning may have been unclear in the Methods section and have added this clarification in the revised submission. We also appreciate your feedback regarding the breath of literature covered and, in the discussion, we proposed to audit specific CAIM journals in future research endeavours.

• Last, I feel it would be helpful to perhaps consider some points in the discussion about the potential need for gathering input from the field of CAIM to identify their needs, concerns, and supports relevant to implementing more OS practices and TOP Guidelines as part of next steps and future research. For example, with qualitative research there have been concerns and relevant points identified in recent years related to the use of OS practices and also implementation of TOP guidelines. Advancement in this area of OS practices in qualitative research has occurred due to explicit scholarly discourse on the topic that includes varied viewpoints as well as recent collaborations between OS experts and experts in the field of qualitative research. I feel this also is relevant when it comes to the needs of particular fields of disciplinary expertise. Although I would agree that we can all benefit from improving our research and practices, there will be nuances and potential impacts specific to particular disciplines and professional areas of expertise. Therefore, future studies that focus on gathering feedback from field experts as part of the ongoing improvement and implementation process of guidelines will only strengthen all fields of study, and perhaps the TOP Guidelines as well.

- We thank you for this suggestion. We, too, find this future direction relevant and meaningful as you have explained. We have added in a short paragraph summarizing this point with appropriate sources cited where needed.

Thank you for the opportunity to review. I appreciate the time of the authors in examining this topic of importance.

Reviewer #2: The manuscript describes an audit conducted to investigate open science practices in complementary, alternative, and integrative medicine (CAIM) journals. It utilized established guidelines and a systematic approach to selecting journals, extracting data, and evaluating open science practices. The results indicate that CAIM journals generally provide minimal guidelines to encourage or require authors to adhere to open science practices.
The statistical analysis employed in the study was descriptive, aligning with the study's aims to examine open science practices in CAIM journals. It involved summarizing journal characteristics and TOP guideline scores using measures like mean, median, range, and standard deviation. This approach is suitable for providing a comprehensive overview of open science practices across this journals.
All relevant data are included in this manuscript or posted on Open Science Framework at the link <https://doi.org/10.17605/OSF.IO/S7G6P>

- We kindly thank this reviewer for providing their feedback on our manuscript.

Recommendations:
Highlighting in the Strengths section of this research the explicit improvement of open science practices in these journals could benefit both researchers and healthcare professionals, as well as patients who use complementary and integrative medicine treatments.

- We appreciate your recommendation. We believe that the Strengths section should focus on methodological strengths, however we do touch on this strength in the second paragraph of the discussion section. Based on your recommendation, we have since added more explicit information on how improvement of open science practices can benefit these individuals.

Explaining in more detail the possible factors that contribute to the low scores of open science practices in complementary and integrative medicine journals, such as barriers they may face: for example, social, cultural, and economic barriers.

- We thank you for this suggestion. We have added more information about cultural barriers that CAIM journals may face with respect to open science implementation.

6. PLOS authors have the option to publish the peer review history of their article ([what does this mean?](https://journals.plos.org/plosone/s/editorial-and-peer-review-process#loc-peer-review-history)). If published, this will include your full peer review and any attached files.

If you choose “no”, your identity will remain anonymous but your review may still be made public.

**Do you want your identity to be public for this peer review?** For information about this choice, including consent withdrawal, please see our [Privacy Policy](https://www.plos.org/privacy-policy).

Reviewer #1: **Yes:** Sondra Stegenga

Reviewer #2: No

=====================================================================

Should your editorial office require any further edits following my most recently submitted submission, please do not hesitate to inform me and we will make these changes as soon as possible. Thank you for your consideration.

Yours sincerely,

Dr. Jeremy Y. Ng, MSc, PhD

Postdoctoral Fellow, Centre for Journalology, Ottawa Methods Centre, Ottawa Hospital Research Institute, Ottawa, Canada

Research Associate, Institute of General Practice and Interprofessional Care, University Hospital Tübingen, Tübingen, Germany & Robert Bosch Center for Integrative Medicine and Health, Bosch Health Campus, Stuttgart, Germany
